# Connexin 26 (GJB2) gene mutations linked with autosomal recessive non-syndromic sensor neural hearing loss in the Iraqi population

**DOI:** 10.25122/jml-2021-0152

**Published:** 2021

**Authors:** Anwar Madlool Al-janabi, Habeeb Shuhaib Ahmmed, Salih Mahdi Al-Khafaji

**Affiliations:** 1. Department of Clinical Chemistry, College of Medicine, University of Kufa, Najaf, Iraq; 2. Department of Otolaryngology, College of Medicine, University of Kufa, Najaf, Iraq; 3. Department of Anatomy & Histology, College of Medicine, University of Kufa, Najaf, Iraq

**Keywords:** GJB2, Connexin 26, mutations, PCR-RFLP

## Abstract

Deafness is a total or partial hearing loss that may appear at any age and with different degrees of severity. Approximately 50% of hearing loss have a genetic origin, and among them, non-syndromic sensorineural deafness represents about 70% of the cases. From them, 80% correspond to autosomal recessive inheritance deafness. Autosomal recessive deafness was not studied enough at the molecular level in Iraq. This study aimed to verify the frequency of three GJB2 mutations in non-syndromic sensorineural deafness in the Iraqi population. The current case-control study was conducted from January 2018 to January 2020. The study included 95 deafness patients (55 males and 40 females) and 110 healthy control group. Age and sex were matched between the two groups. In order to detect c.35delG, 235delC, and 167delT mutations in the GJB2 gene, we employed the PCR-RFLP technique. The c.35delG was the main frequent mutation encountered with the GJB2 gene among patients with autosomal recessive non-syndromic sensorineural hearing loss. Among them, 35 (36.8%) were homozygous, 40 (42.1%) were heterozygous, and 20 (21.1%) were wild genotypes. The second-degree mutation in the GJB2 gene was c.235delC mutation, which from the 95 deaf patients, there were 20 (21.1%) with homozygous, 33 (34.7%) heterozygous, and 42 (44.2%) wild genotypes. None of the 95 deaf patients showed the c.167delT mutation, and no mutations appeared in the control group. Our data concluded that the GJB2 c.35delG and c.235delC gene mutations were the main cause of autosomal recessive non-syndromic sensorineural hearing loss in the Iraqi deaf population.

## Introduction

Deafness is the partial or complete loss of hearing capacity, affecting 6 to 8% of the developed country’s population at any age and with varying severity. The consequences to the individual and society are highly influenced by the age of appearance and its severity. When occurring early in childhood and comprising a severe defect, it causes profound limitations for learning to speak and subsequent cognitive and psychosocial disorders. In the elderly, it affects the quality of life due to the isolation of the individual affected [1]. Congenital hearing loss is the most common form of sensory impairment in humans, with approximately 1/1000 infants being born with a severe hearing deficit [2]. Hereditary hearing loss is divided into two types: syndromic and non-syndromic. The syndromic type is associated with other characteristics in clinical features with deafness which accounts for 30% of hereditary congenital deafness. On the other hand, the non-syndromic type, deafness, is only a clinical appearance representing the other 70% [3]. Regarding the non-syndromic hearing loss (NSHL), autosomal recessive is the most common form accounting for 75% to 85% of the cases [4–5]. More than 100 genes have been linked to NSHL; however, the gap junction protein connexin 26 (GJB2) has the highest rate of mutations. Connexin 26 (GJB2) is a large family of proteins that forms gap junctions close to every cell type. Multimeric connexons make up this junction, which allows molecules to flow from one cell to the next. Connexin, which differs in cell specificity and gating features, makes up the connexons [6]. The gene encoding gap junction protein connexin 26 (GJB2) is located on chromosome 13q11-12 and reported to have up to 50% mutations in all patients from different populations. Although numerous different mutations were recognized (35delG, 235delC, 167delT, V37I, 109G-A etc), 235delC was the most common GJB2 mutation in East Asian populations [7–12].

No molecular study of the connexin 26 (GJB2) genes was reported in Iraqi patients with non-syndromic sensorineural hearing loss. Therefore, this study aimed to investigate the common c.35delG, c.235delC, and c.167delT mutations in the connexin 26 (GJB2) genes in Iraqi deafness patients.

## Material and Methods

Ninety-five patients diagnosed with bilateral non-syndromic sensorineural hearing loss were studied from January 2018 to January 2020. The subjects were 55 males and 40 females from the middle Euphrates region of Iraq, and their ages ranged from 11–40 years old. Among them, 75 were sporadic cases of hearing loss, compatible with recessive inheritance, while the other 20 probands were from families with more than one sib of non-syndromic hearing loss. Age and gender matched with the control group, which consisted of 110 voluntary healthy individuals unrelated to patients. The sample size was calculated using the online software OSSE [13]. Individuals with acquired hearing loss related to environmental causes and syndromic hearing loss were excluded from this study. The assessment included a complete case history using Pure Tone Audiometry and physical examination. The inclusion criteria were the final findings of the examinations with non-syndromic sensorineural hearing loss patients.

### Audiology

All individuals underwent pure-tone audiometry by a diagnostic audiometer (Siemens Danplex DA 74 Clinical Diagnostic Audiometer, USA) in a soundproof room. Pure-tone averages of more than 25 dBHL (mean dBHL at 500, 1000, and 2000 Hz) were defined as hearing loss according to the hearing loss classification [14]. The results revealed that the degree of hearing impairment ranged from moderate to profound in studied subjects (Table 1).

**Table 1.** Degree of hearing loss classification based on the hearing threshold.

**Degree of Hearing Loss Tone Average**	**Hearing Loss range(dB HL)**
**Normal**	-10 to 15
**Slight**	16 to 25
**Mild**	26 to 40
**Moderate**	41 to 55
**Moderately severe**	56 to 70
**Severe**	71 to 90
**Profound**	91 to equipment limits

### Mutation Analysis

Venous blood (1ml) was collected from all individuals who participated in this study. Genomic DNA was isolated and purified from peripheral blood lymphocytes using ReliaPrep™ Blood gDNA Miniprep System (Promega) according to the manufacturer’s protocol. Extracted DNA was used in screening for the selected mutations. PCR-Restriction fragment length polymorphism (PCR-RFLP) analyses were performed to detect three GJB2 common mutations previously described by other investigators and indicated in Table 2. PCR primers and restriction enzymes were purchased from Biolabs (USA). The amplification of the GJB2 gene was performed using 100–200 ng genomic DNAs in a 25 μl mixture containing 50mM KCl, 10mM Tris HCl (pH 8,3), 1.5mM MgCl2, 200M dNTPs, and 1.25U tag DNA Polymerase (Promega, USA) and 10pmol of each primer. The PCR was done by incubation at 94°C for 15sec, followed by 35 cycles of 94°C for 15 sec., 55°C for 15sec. and 72°C for 90 sec. The PCR products were digested with BstI, ApaI, and MwoI enzymes to search for the connexin 26 (GJB2) variations c.35delG, c.235delC and c.167delT, respectively. Wild-type PCR products were cut by the enzymes in c.35delG and c.235delC genes and produced double bands in gel electrophoresis. 167delT mutated gene was uncut and produced a single band when analyzed by electrophoresis in a 2% agarose gel containing ethidium bromide, and the results were recognized by the gel documentation system. Statistical analyses were performed using the SPSS windows software 20 (SPSS Inc., Chicago, IL) 

**Table 2.** Mutations and parameters for PCR-RFLP.

Primer sequence	Mutation	PCR Product (bp)	Restriction Enzyme	Allele size (bp)	Ref.
**F:TCTTTTCCAGAGCAAACCGC** **R:GCTGGTGGAGTGTTTGTTCACA**	c.35delG	89	BstI	Wild: 69+20 Hetero: 89+69 Homo: 89	[19]
**F: TGTGTGCATTCGTCTTTTCCAG** **R:GGTTGCCTCATCCCTCTCAT**	c.235delC	722	ApaI	Wild: 451+277 Hetero: 722+451+277 Homo: 722	[28]
**F: GATTGGGGCACGCTGCA** **R: CCCTTGATGAACTTCCTCTTCT**	c.167delT	322	MwoI	Wild: 322 Hetero: 322+161 Homo: 161	[2]

bp – base pair; F – Foreword; R – Reverse; Ref – Reference.

## Results

The current study involved three mutations in Connexin 26 (GJB2) gene-related with non-syndromic autosomal recessive deaf-mute among 95 patients. 55 (57.9%) were males, and 40 (42.1%) were females. According to the results of our survey, endogamy marriage between parents of deaf patients was more common (80%), and there was a family history of deafness in either the mother’s family (39%) or the father’s family (42%). However, 89 out of 95 (94%) of the patients’ parents have normal hearing. The presented data for five of the remaining sex patients appeared that their paternal and maternal grandparents have normal hearing. Hence, from the interview outcomes, all patients were considered as having autosomal recessive non-syndromic hearing loss. A total of 95 deaf patients with ARNSHL who were recruited in this study were included 75 simplex proband’s which refers to sporadic patients whose families have no one suffering from hearing loss, and 20 multiplex proband’s which means that there is at least one first or second degree deaf relative in their families. Clinical characteristics of deaf patients and hearing tests confirmed that the level of hearing loss was severe to profound in 91 patients; the remaining 4 showed a moderate hearing loss, as shown in Table 3. The present study revealed that c.35delG was the most frequently encountered mutation in the GJB2 gene. From 95 deaf patients, there were 35 (36.8%) with homozygous genotype, 40 (42.1%) with heterozygous genotype, while the remaining 20 (21.1%) had wild genotype as shown in Figure 1.

**Table 3.** Clinical characteristics of deafness patients.

Parameters	Simplex proband’s n=75	Multiplex proband’s n=20
**Sex**		
**Male 55**	42	13
**Female 40**	33	7
**Age at the test**		
**6–18 years**	40	9
**19–40 years**	35	11
**Severity of hearing loss**		
**Mild**	0	0
Moderate	4	0
**Severe**	11	3
**Profound**	60	17

**Figure 1.**
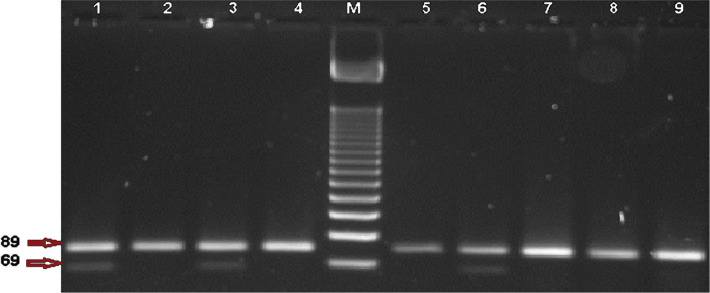
Restriction analysis by BstI enzyme for PCR product of c.35delG mutation in GJB2 gene in 3% agarose gel electrophoresis. Lane M: DNA Ladder 50bp, Lane 1, 3, and 6 are heterozygous genotype (89+69 bp), and Lane 2, 4, 5,7, 8, and 9 homozygous genotypes (89 bp)of mutant alleles.

**Figure 1.**
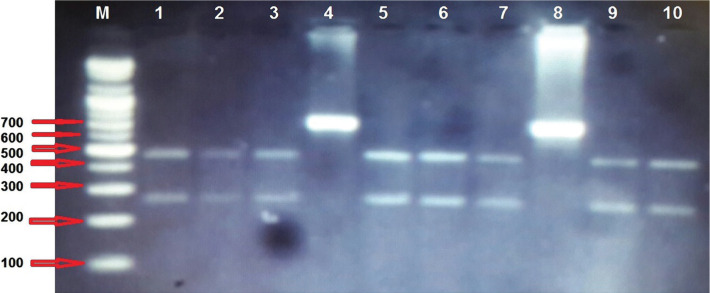
Restriction analysis by ApaI enzyme for PCR product of c.235delC mutation in GJB2 gene in 2% agarose gel electrophoresis. Lane M: DNA Ladder. Lane 1, 2, 3, 5, 6, 7, 9, and 10 are wild genotypes (451+277 bp), Lane 4, and 8 are homozygous genotypes (722 bp) of mutant alleles.

On the other hand, the c235delC mutation was the second-degree mutation in the GJB2 gene from deaf patients; there were 20 (21.1%) patients with homozygous genotype, 33 (34.7%) heterozygous genotype, and 42 (44.2) wild genotypes for the tested mutation as shown in Figure 2. None of the 95 deaf patients showed the c.167delT mutation, and no mutations appeared in the control group. The frequency of the detected mutations is summarized in Table 4.

**Table 4.** Frequency of the GJB2 mutations in deafness patients.

**GJB2 mutation**	**Effect**	**Genotype/Frequency (%)**				**Allele frequency**
		**Wild**	**Heterozygous**	**Homozygous**	**Total affected**	
**c.35delG**	frame shift	20 (21.1%)	40 (42.1%)	35 (36.8%)	75 (78.9%)	0.42%
**c.235delC**	frame shift	42 (44.2%)	33 (34.7%)	20 (21.1%)	53 (55.8%)	0.61%
**c.167delT**	frame shift	Not detected					-

## Discussion

The GJB2 gene encoding the gap-junction protein Connexin 26 caused different forms of hearing impairment, in particular autosomal recessive non-syndromic hearing impairment. The mutations of this gene as heterozygous or homozygous forms influence the activities of the gap junctions and thus prevent the transformation of the mechanical signal into an electrical signal. Hence deafness was installed as bilateral and symmetrical, most often severe or profound [7]. Mutations in GJB2 are the most common cause of moderate-to-profound congenital inherited hearing impairment in numerous populations [10]. In Iraq, there were no details about this type of mutation except for Jarada *et al.* [15], who studied other variant mutations in the GJB2 gene of Iraqi people who were resident in the Jordan kingdom. The distribution of Connexin 26 (GJB2) mutations varies greatly within ethnic groups, GJB2 c.35delG variant being present in 60% of Caucasians, Northern Europeans, and Turkish people with hereditary hearing loss [16–17]. Therefore, we performed this case-control study to investigate the role of c.235delC, c.35delG, and.167delT mutations in the GJB2 and their interaction with other environmental factors to the susceptibility of this condition. This study investigated three GJB2 mutations in NSHL from the middle Euphrates region of Iraq. Although the distribution of GJB2 mutations differs widely between ethnicities, the results revealed that the c. 35delG was evident in 75 (78.9%), which is consistent with the 70% prevalence of the c.35delG mutation recorded and 74% reported by Yenitse *et al.* in patients from Cuba and Spain [18]. Furthermore, 60% of Northern Europeans, Caucasians, and Turks with hereditary deafness had the GJB2 c.35delG mutation [16]. The c.35delG was the most common mutations in GJB2 compared to its frequency in other Arabic groups. GJB2 mutations were found in 40% of Algerians, 33.3% in Lebanon, 23% in Palestine, 17% in Tunisia, and 16.9% in Jordanians. However, this mutation is the commonly encountered GJB2 mutation in Caucasians; it is very rare in Asian patients [19–20]. 

Another less frequent frameshift mutation, c.235delC, was reported in this study, which was detected in 53 (55.8%) of all ARNSHL patients, including 20 (21.1%) homozygous and 33 (34.7%) heterozygous genotypes of mutant alleles. The c.235delC mutation was the most common mutation that caused premature protein termination in patients suffering from hearing loss in East and Southeast Asia, while lower frequencies were recorded in Oceania and Europe [21–23]. It was also found in Japanese, Korean, and Mongolian populations [24–26]. Therefore, a mutation in GJB2 is an important contributor to recessively inherited NSHL in the Chinese population, as appeared in other ethnic groups [27]. The third mutation, c.167delT in the GJB2 gene, which did not appear in our patients, is predominant in Ashkenazi Jews [28]. Also, this mutation was detected in a group of Palestinians from Bethlehem [2], indicating that the variation of GJB2 mutant allele frequency may also be marked in groups of similar populations. Several studies demonstrate a shared origin of the mutation in GJB2, such as the 167delT mutation in Ashkenazi Jews and the R143W mutation in numerous families in a Ghanaian village [28]. The variation between different studies in type of GJB2 mutations and terms of frequency associated with NSHL may be due to several reasons that include sample size (large sample size increase the chance of detecting rare mutations), selection criteria of the patients investigated, the accuracy of genotyping method that employed and consanguineous marriage rate.

These data support the usage of screening programs in the future, for example, suitable genetic tests, as a valuable complement to audiometric screen for identifying neonates with heritable congenital hearing impairment in non-endogamous Iraqi. Applying this test could facilitate the earlier habilitation in a substantial percentage of deaf infants and ultimately provide parents with appropriate prognostic for genetic counsel, specific clinical diagnosis, and relevant medical administration information.

## Conclusion

We concluded from the current study that Connexin 26 (GJB2) c.35delG and c.235delC gene mutations were the causative agent for congenital autosomal recessive hearing loss in the Iraqi population. These data can support the screening programs of audiometric recognizing neonates with congenital inherited hearing loss in Iraqi endogamy parents.

## Acknowledgments

### Conflict of interest

The authors declare that there is no conflict of interest.

### Ethical approval

This case-control study was approved by the medical ethics committee in the Faculty of Medicine/Kufa University (ID: MEC-21 on May 21, 2018).

### Consent to participate

Informed consent was obtained from all participants’ relatives and parents of patients younger than 18 years.

### Personal thanks

The authors want to thank the patients for their cooperation during the time of the study. Also, our thank appreciation extends to the staff of ENT Departments from the Middle Euphrates region of Iraq for their support during sample collection, mainly ENT Department in Al-Sadder hospital in Najaf/Iraq. Finally, the authors want to thank Dr. Saad Mahbobah for his support.

### Authorship

AMA-j is the corresponding author and was involved in collecting data, manuscript concept, writing, genetic analysis, submitted manuscript, revision, and galley proof. HAS was involved in data collection and assessed the audiology of hearing. SMA-k was involved in data collection, manuscript concept, writing, and genetic analysis.
